# Interactive Effects of Dietary Crude Protein and Methionine Levels on Growth Performance, Carcass Quality and Blood Biochemistry of Japanese Quail During Starter and Grower Phases

**DOI:** 10.1002/vms3.71301

**Published:** 2026-10-28

**Authors:** Elyas Piroz‐Maleki, Mehdi Taginejad‐Rodbaneh, Ruhollah Kianfar, Sima Moghaddaszadeh‐Ahrabi, Abolfazl Ghorbani

**Affiliations:** ^1^ Department of Animal Science, Ta.C. Islamic Azad University Tabriz Iran; ^2^ Department of Animal Science, Faculty of Agriculture University of Tabriz Tabriz Iran; ^3^ Department of Animal Science, Shab.C. Islamic Azad University Shabestar Iran

**Keywords:** blood parameters, carcass traits, dietary protein, growth performance, Japanese quail, methionine requirement, nonlinear regression, thyroid hormones

## Abstract

**Background:**

This study evaluated the interactive effects of dietary crude protein (CP) and methionine (Met) on growth performance, carcass traits, blood biochemistry, thyroid hormones and liver enzymes in Japanese quail during the starter (0–14 days) and grower (14–35 days) phases.

**Methods:**

A total of 1,350 mixed‐sex chicks were used in a completely randomised 3 × 5 factorial design, testing three CP levels (22%, 24%, 26% of diet starter; 21%, 23%, 25% of diet grower) and five Met levels (0.30%–0.70% of diet). Performance traits, carcass characteristics, blood indices, thyroid hormones (T3 and T4) and liver enzymes (AST and ALT) were measured, and nonlinear regression models were used to estimate optimal Met requirements.

**Results:**

Raising starter‐phase CP to 26% significantly improved weight gain (+4.1%), feed conversion ratio (−4.1%), and carcass yield (+1.5%). Methionine supplementation up to 1.30% further enhanced these traits, with optimal Met levels ranging from 0.50%–0.70% depending on CP concentration. In the grower phase, the combination of 25% CP and 1.30% Met produced the highest male weight gain (161.84 g) and breast yield (29.88%), while females showed the highest breast yield (30.90%) at the same levels, indicating sex‐specific responses. Increasing Met reduced blood glucose, triglycerides, and cholesterol while raising HDL, with most blood parameters showing significant CP × Met interactions. Thyroid hormones rose with higher CP, though T3 declined with Met supplementation; both factors also influenced liver enzyme activity.

**Conclusions:**

These findings confirm a synergistic relationship between dietary protein and methionine, supporting 24%–26% CP with 0.50%–0.70% Met in the starter phase and 23%–25% CP with appropriate Met levels in the grower phase for modern quail strains—offering updated recommendations beyond the older Nutrient Requirement Council (NRC) guidelines for commercial production.

## Graphical abstract

Dietary crude protein and methionine interact significantly in Japanese quail. Optimal methionine ranges from 0.50%–0.70% depending on protein level and phase. Best grower performance occurs at 25% CP with 1.30% Met, yielding 161.84 g male weight gain. Females show superior breast yield (30.90%). Findings support phase‐ and sex‐specific feeding and updated NRC (1994) recommendations.

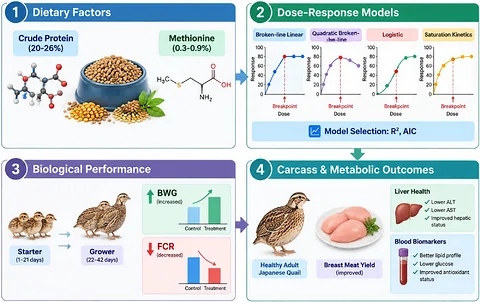

## 1 Primary Audience

This study is primarily intended for poultry nutritionists, animal science researchers and feed formulation specialists working on Japanese quail or small‐game bird production. It provides actionable, phase‐specific recommendations on optimising dietary protein and methionine levels that can be directly applied by commercial quail producers, integrators and nutrition consultants to improve growth performance, carcass quality, feed efficiency and metabolic health while reducing production costs.

The manuscript will also be of interest to academics and graduate students in poultry science, as well as to extension professionals and policymakers seeking science‐based strategies for more sustainable, precise feeding programs in intensive quail farming systems.

## 2 Description of Problem

Japanese quail (*Coturnix japonica*) is a highly productive poultry species valued for its contribution to high‐quality animal protein production. Its short production cycle, rapid growth rate, early sexual maturity, high egg output (280–300 eggs/year) and relative disease resistance make it an attractive option for both meat and egg producers (Minvielle 2004; NRC 1994). Owing to its adaptability to intensive systems, minimal space requirements, low initial investment, and quick returns, quail farming has become an economically viable source of protein, particularly in developing countries.

Nutrition is central to efficient, sustainable poultry production, as feed costs can account for up to 70% of total production expenses (Leeson and Summers 2005), making precise dietary formulation essential for optimising performance while limiting costs and environmental impact. In corn–soybean meal‐based diets, methionine—a sulphur‐containing amino acid—is typically the first limiting amino acid, and its adequate supplementation is critical for growth, feed efficiency, carcass quality and egg production (Baker 2009).

Beyond protein synthesis, methionine serves as a precursor of S‐adenosylmethionine (SAM) for methylation reactions and supports the synthesis of sulphur‐containing compounds such as cysteine, glutathione and taurine (Martín‐Venegas et al. 2006; Bunchasak 2009). These functions extend to gene expression, lipid metabolism, immune response and antioxidant defence, particularly under oxidative stress from high‐fat or high‐energy diets (Waterland 2006; Surai et al. 2019). Japanese quail may have higher methionine requirements than other poultry species due to distinct metabolic rates and feed intake patterns (Shim and Vohra 1984).

Dietary protein and energy levels strongly influence methionine requirements. The ideal protein concept emphasises supplying essential amino acids in balanced proportions—typically relative to lysine—to optimise nitrogen utilisation and reduce excretion (Emmert and Baker 1997). Higher‐protein diets naturally supply more methionine from intact protein sources such as soybean meal and other feedstuffs. In contrast, lower‐protein diets often lack sufficient methionine from these natural sources and therefore require supplementation with crystalline (synthetic) methionine to maintain optimal amino acid ratios (Esteve‐Garcia and Austic 1993; Aletor et al. 2000). Energy density adds further complexity by affecting feed intake: high‐energy diets may reduce intake and, if not properly balanced, limit total methionine consumption (Dozier et al. 2008). In broilers, the optimal methionine‐to‐lysine ratio has been shown to vary with dietary energy density, reflecting interactions between sulphur amino acids and energy metabolism (Liu et al. 2015; Conde‐Aguilera et al. 2013).

Reported methionine requirements for Japanese quail range from approximately 0.35% to 0.50% of the diet, varying with protein level, strain, age, sex and production stage (Murakami et al. 1993; Baylan et al. 2006). However, NRC (1994) recommendations, based on older data, may not reflect the genetic potential of modern quail strains or their interaction with current protein and energy levels. Environmental stressors, such as heat stress, can further increase methionine requirements due to its antioxidant role, while genetic selection for faster growth in modern lines likely alters nutrient utilisation (Havenstein et al. 2003). The studies in broilers indicate that methionine can be converted into cysteine at rates ranging from 27% to 43%, with an optimal Met: Cys ratio of approximately 52:48 on a total sulphur amino acid basis (Pacheco et al. 2018)

Despite this, precise methionine requirements for Japanese quail under varying protein and energy levels—especially across distinct growth phases—remain poorly defined, as most prior research has focused on broilers or limited quail life stages. Without accounting for these interactions, birds cannot achieve their full genetic potential. Therefore, this study investigated the interactive effects of graded dietary crude protein levels (22%, 24% and 26% for days 0–14; 21%, 23% and 25% for days 14–35) and methionine levels (0.30%, 0.40%, 0.50%, 0.60% and 0.70%) on growth performance, feed efficiency, carcass traits, blood biochemistry, thyroid hormones and liver enzyme activity in Japanese quail. The primary objective was to identify optimal protein and methionine levels that maximise productive performance while minimising metabolic stress; secondary objectives included evaluating sex‐specific responses and assessing whether current NRC recommendations require updating for modern quail genotypes.

## 3 Material and Methods

### 3.1 Birds, Diets and Management

This experiment was conducted to determine the optimal dietary level of methionine for Japanese quails up to five weeks of age.

A total of 1350 quails of both sexes were used. Birds were fed formulated diets based on the nutritional requirements for quails (NRC 1994). Feed and water were provided ad libitum throughout the experimental period. The birds were allocated to a 3×5 factorial experiment in a completely randomised design, with 6 replications per treatment and 15 quails per replicate. The trial lasted for five weeks. The composition and nutrient content of the experimental diets are provided in Tables 1 and 2. At the beginning of the grower phase (14 days of age), birds were sexed based on plumage dimorphism, and males and females were monitored separately thereafter.

**TABLE 1:** Ingredients of experimental diets for 0–14 days old birds.

Protein level (%)	22					24					26				
Methionine level (%)	0.3	0.4	0.5	0.6	0.7	0.3	0.4	0.5	0.6	0.7	0.3	0.4	0.5	0.6	0.7
**Ingredients** ^a^	(% as fed)														
Gluten meal	2.93	2.93	2.93	2.93	2.93	3.12	3.12	3.12	3.12	3.12	3.3	3.3	3.3	3.3	3.3
Corn, grain	59.31	59.31	59.31	59.31	59.31	57.32	57.32	57.32	57.32	57.32	55.32	55.32	55.32	55.32	55.32
Soybean meal ‐44%	28.11	28.11	28.11	28.11	28.11	28.23	28.23	28.23	28.23	28.23	28.34	28.34	28.34	28.34	28.34
Canola oil	0.5	0.5	0.5	0.5	0.5	0.5	0.5	0.5	0.5	0.5	0.5	0.5	0.5	0.5	0.5
Corn starch	2	1.948	1.896	1.844	1.792	2	1.948	1.896	1.844	1.792	2	1.948	1.896	1.844	1.792
Dical. Phos.	2.04	2.04	2.04	2.04	2.04	2.05	2.05	2.05	2.05	2.05	2.06	2.06	2.06	2.06	2.06
Bicarbonate sodium	0.24	0.24	0.24	0.24	0.24	0.24	0.24	0.24	0.24	0.24	0.24	0.24	0.24	0.24	0.24
Limestone	1.22	1.22	1.22	1.22	1.22	1.21	1.21	1.21	1.21	1.21	1.21	1.21	1.21	1.21	1.21
Common salt	0.22	0.22	0.22	0.22	0.22	0.22	0.22	0.22	0.22	0.22	0.22	0.22	0.22	0.22	0.22
Vit^b^ and min^c^ premix	0.5	0.5	0.5	0.5	0.5	0.5	0.5	0.5	0.5	0.5	0.5	0.5	0.5	0.5	0.5
DL‐methionine	0	0.102	0.204	0.306	0.408	0	0.102	0.204	0.306	0.408	0	0.102	0.204	0.306	0.408
L‐lysine HCl	0.46	0.46	0.46	0.46	0.46	0.46	0.46	0.46	0.46	0.46	0.46	0.46	0.46	0.46	0.46
L‐threonin	0.37	0.37	0.37	0.37	0.37	0.37	0.37	0.37	0.37	0.37	0.37	0.37	0.37	0.37	0.37
L‐valine	0.04	0.04	0.04	0.04	0.04	0.04	0.04	0.04	0.04	0.04	0.04	0.04	0.04	0.04	0.04
L‐glutamic	1.87	1.82	1.77	1.72	1.67	3.56	3.51	3.46	3.41	3.36	5.25	5.2	5.15	5.1	5.05
L‐isoleucine	0.19	0.19	0.19	0.19	0.19	0.19	0.19	0.19	0.19	0.19	0.19	0.19	0.19	0.19	0.19
Total	100	100	100	100	100	100	100	100	100	100	100	100	100	100	100
**Components**															
AMEn (Mcal/kg)	2.95	2.95	2.95	2.95	2.95	2.95	2.95	2.95	2.95	2.95	2.95	2.95	2.95	2.95	2.95
CP%	22	22.1	22.2	22.2	22.3	24	24.1	24.2	24.2	24.3	26	26.1	26.2	26.2	26.3
Calcium%	1	1	1	1	1	1	1	1	1	1	1	1	1	1	1
Avail. phosphorus%	0.5	0.5	0.5	0.5	0.5	0.5	0.5	0.5	0.5	0.5	0.5	0.5	0.5	0.5	0.5
Potassium%	0.75	0.75	0.75	0.75	0.75	0.75	0.75	0.75	0.75	0.75	0.75	0.75	0.75	0.75	0.75
Chlorine%	0.2	0.2	0.2	0.2	0.2	0.2	0.2	0.2	0.2	0.2	0.16	0.16	0.16	0.16	0.16
Sodium%	0.16	0.16	0.16	0.16	0.16	0.18	0.18	0.18	0.18	0.18	0.18	0.18	0.18	0.18	0.18
Choline%	1.3	1.3	1.3	1.3	1.3	1.3	1.3	1.3	1.3	1.3	1.3	1.3	1.3	1.3	1.3
Arg%	1.26	1.26	1.26	1.26	1.26	1.26	1.26	1.26	1.26	1.26	1.26	1.26	1.26	1.26	1.26
Lys%	1.3	1.3	1.3	1.3	1.3	1.3	1.3	1.3	1.3	1.3	1.3	1.3	1.3	1.3	1.3
Met%	0.3	0.4	0.5	0.6	0.7	0.3	0.4	0.5	0.6	0.7	0.3	0.4	0.5	0.6	0.7
TSAA	0.62	0.72	0.82	0.92	1.02	0.62	0.72	0.82	0.92	1.02	0.62	0.72	0.82	0.92	1.02
Tyr%	0.81	0.81	0.81	0.81	0.81	0.84	0.84	0.84	0.84	0.84	0.82	0.82	0.82	0.82	0.82
Thr%	1.1	1.1	1.1	1.1	1.1	1.1	1.1	1.1	1.1	1.1	1.1	1.1	1.1	1.1	1.1
Val%	0.95	0.95	0.95	0.95	0.95	0.95	0.95	0.95	0.95	0.95	0.95	0.95	0.95	0.95	0.95
Trp%	0.22	0.22	0.22	0.22	0.22	0.22	0.22	0.22	0.22	0.22	0.22	0.22	0.22	0.22	0.22
Leu%	1.80	1.80	1.80	1.80	1.80	1.7	1.7	1.7	1.7	1.7	1.69	1.69	1.69	1.69	1.69

^a^ Based on NRC (1994) feed composition tables.

^b^ Vitamin premix provided the following per kilogram of diet. vitamin A, 5,500 IU (as all trans‐retinyl acetate); cholecalciferol, 1,100 IU; vitamin E, 11 IU (as all‐rac‐α‐tocopherol acetate); riboflavin, 4.4 mg; Ca pantothenate, 12 mg; nicotinic acid, 44 mg; choline Cl, 220 mg; vitamin B12, 6.6 µg;vitamin B6, 2.2 mg; menadione, 1.1 mg (as menadione sodium bisulfitecomplex); folic acid, 0.55 mg; d‐biotin, 0.11 mg; thiamine, 1.1 mg (asthiamine mononitrate); ethoxyquin, 125 mg.

^c^ Provided per kilogram of diet. Mn (from MnSO4_H2O), 100 mg; Zn (from ZnSO4_7H2O), 100 mg; Fe, (from FeSO4_7H2O), 50 mg; Cu (from CuSO4_5H2O), 10 mg; I (from Ca (IO3)2_H2O), 1 mg.

**TABLE 2:** Ingredients of experimental diets for 15–35 days old birds.

Protein level (%)	21					23					25				
Methionine level (%)	0.3	0.4	0.5	0.6	0.7	0.3	0.4	0.5	0.6	0.7	0.3	0.4	0.5	0.6	0.7
**Ingredients** ^1^	(% as fed)														
Gluten meal	2.82	2.82	2.82	2.82	2.82	3	3	3	3	3	3.19	3.19	3.19	3.19	3.19
Corn, grain	60.49	60.49	60.49	60.49	60.49	58.53	58.53	58.53	58.53	58.53	56.54	56.54	56.54	56.54	56.54
Soybean meal ‐44%	28.04	28.04	28.04	28.04	28.04	28.15	28.15	28.15	28.15	28.15	28.27	28.27	28.27	28.27	28.27
Canola oil	0.53	0.53	0.53	0.53	0.53	0.5	0.5	0.5	0.5	0.5	0.5	0.5	0.5	0.5	0.5
Corn starch	2	1.948	1.897	1.846	1.795	2	1.948	1.897	1.846	1.795	2	1.948	1.897	1.846	1.794
Dical. Phos.	1.59	1.59	1.59	1.59	1.59	1.6	1.6	1.6	1.6	1.6	1.61	1.61	1.61	1.61	1.61
Bicarbonate sodium	0.34	0.34	0.34	0.34	0.34	0.34	0.34	0.34	0.34	0.34	0.34	0.34	0.34	0.34	0.34
Limestone	1.48	1.48	1.48	1.48	1.48	1.47	1.47	1.47	1.47	1.47	1.47	1.47	1.47	1.47	1.47
Common salt	0.12	0.12	0.12	0.12	0.12	0.12	0.12	0.12	0.12	0.12	0.12	0.12	0.12	0.12	0.12
Vit^2^ and min^3^ premix	0.5	0.5	0.5	0.5	0.5	0.5	0.5	0.5	0.5	0.5	0.5	0.5	0.5	0.5	0.5
DL‐methionine	0	0.102	0.204	0.306	0.408	0	0.102	0.204	0.306	0.408	0	0.102	0.204	0.306	0.408
L‐lysine HCl	0.46	0.46	0.46	0.46	0.46	0.46	0.46	0.46	0.46	0.46	0.46	0.46	0.46	0.46	0.46
L‐threonin	0.37	0.37	0.37	0.37	0.37	0.37	0.37	0.37	0.37	0.37	0.37	0.37	0.37	0.37	0.37
L‐valine	0.04	0.04	0.04	0.04	0.04	0.04	0.04	0.04	0.04	0.04	0.04	0.04	0.04	0.04	0.04
L‐glutamic	1.02	0.97	0.919	0.868	0.817	2.71	2.66	2.609	2.558	2.507	4.4	4.35	4.299	4.248	4.198
L‐isoleucine	0.19	0.19	0.19	0.19	0.19	0.19	0.19	0.19	0.19	0.19	0.19	0.19	0.19	0.19	0.19
Total	100	100	100	100	100	100	100	100	100	100	100	100	100	100	100
**Components**															
AMEn (Mcal/Kg)	2.95	2.95	2.95	2.95	2.95	2.95	2.95	2.95	2.95	2.95	2.95	2.95	2.95	2.95	2.95
CP %	21	21.09	21.06	21.03	21	23	23.09	23.06	23.03	23	25	25.09	25.06	25.03	25.11
Calcium %	1	1	1	1	1	1	1	1	1	1	1	1	1	1	1
Avail. phosphorus %	0.42	0.42	0.42	0.42	0.42	0.42	0.42	0.42	0.42	0.42	0.42	0.42	0.42	0.42	0.42
Potassium %	0.761	0.761	0.761	0.761	0.761	0.758	0.758	0.758	0.758	0.758	0.755	0.755	0.755	0.755	0.755
Chlorine %	0.2	0.2	0.2	0.2	0.2	0.2	0.2	0.2	0.2	0.2	0.2	0.2	0.2	0.2	0.2
Sodium %	0.16	0.16	0.16	0.16	0.16	0.16	0.16	0.16	0.16	0.16	0.16	0.16	0.16	0.16	0.16
Choline %	1.37	1.37	1.37	1.37	1.37	1.362	1.362	1.362	1.362	1.362	1.353	1.353	1.353	1.353	1.353
Arg %	1.26	1.26	1.26	1.26	1.26	1.26	1.26	1.26	1.26	1.26	1.261	1.261	1.261	1.261	1.261
Lys %	1.3	1.3	1.3	1.3	1.3	1.3	1.3	1.3	1.3	1.3	1.3	1.3	1.3	1.3	1.3
Met %	0.3	0.4	0.5	0.6	0.7	0.3	0.4	0.5	0.6	0.7	0.3	0.4	0.5	0.6	0.7
TSAA	0.619	0.619	0.619	0.619	0.619	0.618	0.618	0.618	0.618	0.618	0.618	0.618	0.618	0.618	0.618
Tyr %	0.811	0.811	0.811	0.811	0.811	0.813	0.813	0.813	0.813	0.813	0.815	0.815	0.815	0.815	0.815
Thr %	1.1	1.1	1.1	1.1	1.1	1.1	1.1	1.1	1.1	1.1	1.1	1.1	1.1	1.1	1.1
Val %	0.95	0.95	0.95	0.95	0.95	0.95	0.95	0.95	0.95	0.95	0.95	0.95	0.95	0.95	0.95
Trp %	0.22	0.22	0.22	0.22	0.22	0.22	0.22	0.22	0.22	0.22	0.22	0.22	0.22	0.22	0.22
Leu %	1.835	1.835	1.835	1.835	1.835	1.84	1.84	1.84	1.84	1.84	1.845	1.845	1.845	1.845	1.845
Ile %	1	1	1	1	1	1	1	1	1	1	1	1	1	1	1
DCAB (meq/kg)	258.5	258.5	258.5	258.5	258.5	257.7	257.7	257.7	257.7	257.7	257.0	257.0	257.0	257.0	257.0

^1^ Based on NRC (1994) feed composition tables.

^2^ Vitamin premix provided the following per kilogram of diet. vitamin A, 5,500 IU (as all trans‐retinyl acetate); cholecalciferol, 1,100 IU; vitamin E, 11 IU (as all‐rac‐α‐tocopherol acetate); riboflavin, 4.4 mg; Ca pantothenate, 12 mg; nicotinic acid, 44 mg; choline Cl, 220 mg; vitamin B12, 6.6 µg;vitamin B6, 2.2 mg; menadione, 1.1 mg (as menadione sodium bisulfitecomplex); folic acid, 0.55 mg; d‐biotin, 0.11 mg; thiamine, 1.1 mg (asthiamine mononitrate); ethoxyquin, 125 mg.

^3^ Provided per kilogram of diet. Mn (from MnSO4_H2O), 100 mg; Zn (from ZnSO4_7H2O), 100 mg; Fe, (from FeSO4_7H2O), 50 mg; Cu (from CuSO4_5H2O), 10 mg; I (from Ca (IO3)2_H2O), 1 mg.

The lighting program consisted of continuous light (24 h light:0 h dark) during the first 7 days of age, followed by 23 h light:1 h dark from day 8 until the end of the experiment (day 35). Light intensity was maintained at 20–25 lux during the first week and approximately 15–20 lux thereafter at bird level.

Ethical statement. All animals were treated humanely in accordance with the ethical codes and guidelines for animal research in Iran and international veterinary standards. Every effort was made to reduce pain, stress and discomfort to the animals. The methionine level included five dietary levels during each phase: 0.30%, 0.40%, 0.50% (NRC 1994 recommendation), 0.60% and 0.70% methionine. During the 1–14‐day age, three protein levels included 22%, 24% and 26% and during the 14–35 days of age, the other three protein levels 21%, 23% and 25% were tested.

Estimation and measurement of methionine in experimental diets were based on the total threonine content. The composition of each experimental diet was calculated and the methionine content of each treatment diet was analysed and presented in the corresponding tables.

### 3.2 Performance

During the experimental period, performance parameters including body weight gain (BWG), feed intake (FI), mortality rate and feed conversion ratio (FCR) were recorded weekly and calculated daily. To ensure accuracy, birds were fasted for 4 hours before weighing to clear the digestive tract of feed residues. Body weight (BW) was measured at the beginning of the trial and at the end of each experimental period. Weighing was performed in groups, and the average BW was calculated accordingly. To determine daily FI, the amount of feed offered to each replicate was recorded at the beginning of each period (1‐14, 15–35d). The leftover feed was weighed at the end of the same period, and the difference was considered as FI. FCR was calculated by dividing the amount of feed consumed by the total BWG during each experimental period. The feed conversion ratio (FCR) was calculated as the ratio of feed consumed to body weight gain, corrected for mortality (mortality‐corrected FCR).

### 3.3 Carcass Traits

To investigate the characteristics of the carcass at the end of the course, two quails from each repetition were selected based on the average weight of each unit, weighed and euthanised using the CO_2_ gas method (Akbarnejad et al. 2015). After this, the carcass was dissected, and the relative weight of the carcass, liver, bursa, thigh and breast was measured using a digital scale with an accuracy of 0.001 g. The weight of each of these organs was calculated as a percentage of live weight.

### 3.4 Blood Parameter

Blood samples were collected in heparinised tubes (3 mL), kept on ice, and transported to the laboratory. Some blood samples were collected in the non‐heparinised tube (2 mL) and allowed to clot for two hours at 37°C. Uric acid was determined calorimetrically (Pars Azmon kit, Tehran, Iran), and plasma thyroid hormones (T3, T4) were measured by the ELISA method (Pishtaz Tab Zaman Company Kit, Iran). Plasma glucose (GLU), total protein (TP), cholesterol (CHO), triglyceride (TG), aspartate aminotransferase (AST), albumin (Alb), alanine aminotransferase (ALT) and alkaline phosphatase (ALP) levels were determined by a spectrophotometric device (Pars Azmon kit, Tehran, Iran).

### 3.5 Statistical Model

Data were analysed using SAS software (version 9.1). To determine the optimal dietary methionine levels at each protein concentration and to describe the dose‐response relationships, several nonlinear regression models were fitted to the data using NRM.xls 1.4 software (Vedenov and Pesti 2008). The models tested included: Linear, Quadratic, Broken‐Line, Quadratic‐Broken‐Line, Saturation Kinetics, Logistic (3‐ and 4‐parameter), Richards and Gompertz.

The use of multiple models was adopted because nutritional dose‐response relationships in poultry are biologically complex and may exhibit different patterns (linear ascending, diminishing returns, plateau, or sigmoidal) depending on the trait, sex, age and dietary protein level. No single model is universally superior for all response criteria. Comparing multiple models allows for selection of the most appropriate one based on biological plausibility, goodness‐of‐fit (R^2^) and residual diagnostics. This approach is consistent with recommendations and common practice in poultry nutrition research for estimating amino acid requirements (Vedenov and Pesti 2008; Pesti et al. 2009; Mehri et al. 2014). The best‐fitting model for each trait was selected primarily based on the highest R^2^ value, with the Akaike Information Criterion (AIC) and residual patterns considered where applicable. For traits with low R^2^ (< 0.4), interpretations were made cautiously and primarily supported by ANOVA results.

## 4 Results

### 4.1 Performance and Carcass Traits (0–14 Days)

The summary of the main and interactive effects of dietary levels of protein and methionine on performance and carcass traits of Japanese quail in the starter phase (0 to 14 days of age) is presented in Table 3. Increasing dietary protein from 22.00% to 26.00% significantly improved WG from 58.77 g to 61.20 g (*p* < 0.001) and feed conversion ratio FCR from 101.89 to 97.72 (*p* < 0.001). Higher protein levels increased carcass percentage (*p* = 0.01), but did not affect breast percentage (*p* = 0.18). Methionine levels (0.70% to 1.30%) had a significant positive effect on W.G. (*p* < 0.001), increasing from 56.77 g to 61.36 g, and on FCR (*p* < 0.001), increasing from 101.01 to 99.26. Carcass percentage also increased.

**TABLE 3:** Main and interactive effects of protein and methionine amino acid levels on performance and carcass traits in Japanese quail at 0–14 days age.

	Protein %	Methionine %	WG	FI	FCR	LW	Carcass %	Tight %	Breast %
Protein level	22		58.77^b^	101.89^a^	1.74^a^	65.68^b^	72.03	19.43	30.07
	24		60.08^a^	100.36^a^	1.67^b^	69.61^a^	70.13	19.03	29.25
	26		61.20^a^	97.72^b^	1.60^c^	70.58^a^	70.96	19.12	29.16
	*p*‐value		<0.0001	0.00	<0.0001	<0.0001	0.01	0.051	0.18
	SEM		0.32	0.70	0.01	0.54	0.41	0.29	0.16
Methionine level		0.30	56.77^b^	101.01	1.78^a^	64.63^b^	70.05	18.68^b^	28.77^b^
		0.04	59.13^a^	100.31	1.70^b^	68.39^a^	71.13	18.74^b^	28.86^b^
		0.5	61.19^a^	99.59	1.63^c^	70.17^a^	71.55	19.30^a^	29.55^a^
		0.6	61.62^a^	99.79	1.62^c^	69.89^a^	70.60	19.67a	29.98^a^
		0.	61.36^a^	99.26	1.62^c^	70.04^a^	71.89	19.58^a^	30.29^a^
	*p*‐value		<0.0001	0.68	<0.0001	<0.0001	0.11	0.01	0.00
	SEM		0.41	0.90	0.01	0.70	0.52	0.37	0.20
Protein*methionine interaction	22	0.30	54.23^c^	101.80^a^	1.88^a^	61.90^c^	70.80	18.64	28.77
	22	0.40	58.62^b^	101.50^a^	1.73^c^	65.95^b^	72.28	19.04	29.58
	22	1.50	60.20^a^	101.70^a^	1.69^c^	67.53^ab^	72.94	19.72	30.27
	22	0.60	60.60^a^	102.60^a^	1.69^c^	68.27^ab^	71.36	20.22	31.33
	22	0.70	60.20^a^	101.87^a^	1.70^c^	67.70^b^	72.79	19.54	30.38
	24	0.30	57.53^b^	102.37^a^	1.78^b^	65.37^b^	69.86	18.12	28.56
	24	0.40	58.33^b^	100.67^a^	1.73^a^	66.17^b^	69.84	18.32	28.20
	24	0.50	61.80^a^	100.22^a^	1.62^e^	69.47^a^	70.27	19.04	29.16
	24	0.60	61.37^a^	99.70^ab^	1.63^e^	68.53^ab^	69.57	19.60	29.51
	24	0.70	61.35^a^	98.87^ac^	1.61^e^	68.52^ab^	71.13	20.10	30.81
	26	0.30	58.53^b^	98.87^ab^	1.69^d^	65.87^b^	69.50	19.29	28.98
	26	0.40	60.43^a^	98.77^ab^	1.63^e^	67.77^ab^	71.27	18.86	28.80
	26	0.50	61.58^a^	96.87^b^	1.57^f^	68.92^a^	71.44	19.15	29.23
	26	0.60	62.90^a^	97.07^b^	1.54^f^	70.40^a^	70.86	19.18	29.10
	26	0.70	62.53^a^	97.03^b^	1.55^f^	70.20^a^	71.74	19.10	29.68
	*p*‐value		<0.0001	0.00	<0.0001	<0.0001	0.98	0.55	0.08
	SEM		0.71	1.57	0.01	1.20	0.91	0.64	0.35

*Note*: Means with different letters in each column are statistically significantly different (*p* < 0.05).

Abbrevistions: FCR: feed conversion ratio, FI: feed intake, LW: live weight; WG: weight gain.

Nonlinear regression analyses (Table 4) allowed for the selection of the best predictive models for important performance and carcass traits in response to graded levels of methionine (0.40%–1.00%) at fixed protein levels of 22% and 26% during the 0–14 day growth phase. Plateauing or broken‐line responses indicative of nutritional thresholds were identified by selecting models with the greatest coefficient of determination (R^2^) and residual mean square error. The optimal methionine supplementation range was 0.40% to 0.70% for the traits and protein levels. The median requirement for growth parameters (WG and FI) was approximately 0.50%. The median was a little higher (0.53% to 0.60%) for carcass traits. Reduced methionine requirements with protein limitation were observed at 22% protein (e.g., 0.43% for FCR), with lower thresholds. In contrast, 26% protein diets increased optimal levels (e.g., 0.70% for breast yield), indicating beneficial protein × methionine interactions.

**TABLE 4:** Fitting the best model of different methionine levels on performance traits at 0–14 days of age.

Trait	Protein level %	Best model	R^2^	Equation	Optimal Met %
Weight gain	22	RNB model 1	0.5720	y = 61.19 (1 − exp(− 7.47 MET))	0.40
	24	Quadratic broken line	0.6217	y = 70.67 + −82.61MET + 129.45MET^2 (MET ≤ 0.50), y = 61.51 (MET > 0.50)	0.50
	26	RNB Model 2	0.5956	y = 72.15 (1 − exp(‐5.63MET)) + −15.09MET	0.50
Feed intake	22	Quadratic broken line	0.0038	y = 113.80 + −63.70MET + 79.00MET^2 (MET ≤ 0.53), y = 102.23 (MET > 0.53)	0.53
	24	Logistic 4‐parameter	0.1070	y = 96.15 + (239.56 ‐ 96.15) / (1 + (MET / 0.01)^0.91)	0.30
	26	Quadratic broken line	0.0038	y = 113.80 + −63.70MET + 79.00MET^2 (MET ≤ 0.53), y = 102.23 (MET > 0.53)	0.53
Feed conversion ratio	22	Broken line	0.7856	y = 2.31 + −1.43MET (MET ≤ 0.43), y = 1.69 (MET > 0.43)	0.43
	24	Logistic 4‐parameter	0.8350	y = 1.62 + (1.78 ‐ 1.62) / (1 + (MET / 0.41)^19.70)	0.41
	26	Broken line	0.7856	y = 2.31 + −1.43MET (MET ≤ 0.43), y = 1.69 (MET > 0.43)	0.43
Live weight	22	Logistic 4‐parameter	0.3543	y = 67.56 + (0.00 − 67.56) / (1 + (MET / 0.15)^3.46)	0.30
	24	Quadratic	0.3527	y = 41.82 + 112.77MET + −105.89MET^2	0.53
	26	Logistic 4‐parameter	0.3543	y = 67.56 + (0.00 − 67.56) / (1 + (MET / 0.15)^3.46)	0.30
Carcass yield %	22	Quadratic broken line	0.602	y = 55.33 + 78.04MET + −88.32MET^2 (MET ≤ 0.49), y = 72.36 (MET > 0.49)	0.44
	24	Logistic 4‐parameter	0.0820	y = 83.39 + (69.88 − 83.39) / (1 + (MET / 0.72)^87.83)	0.70
	26	Logistic 3‐parameter	0.89	y = 75 / (1 + exp(−10(MET − 0.5)))	0.50
Tight yield %	22	Quadratic	0.3561	y = 14.09 + 19.77MET + −16.81MET^2	0.59
	24	Logistic 4‐parameter	0.4077	y = 20.49 + (18.03 − 20.49) / (1 + (MET / 0.54)^6.13)	0.54
	26	Broken line	0.90	y = 15 + 10MET (MET ≤ 0.6), y = 18 (MET > 0.6)	0.60
Breast yield %	22	Quadratic	0.1998	y = 22.22 + 28.60MET + −23.79MET^2	0.60
	24	Quadratic	0.2437	y = 30.81 + −13.59MET + 19.41MET^2	0.70
	26	Quadratic broken line	0.93	y = 20 + 30MET − 15MET^2 (MET ≤ 0.6), y = 29 (MET > 0.6)	0.60

**TABLE 5a:** Main and interactive effects of dietary crude protein and methionine levels on growth performance of Japanese quail (14–35 days of age).

Protein (%)	Met (%)	WG (g)	FI (g)	FCR	LW total (g)	LW female (g)	LW male (g)
**Main effect of protein**							
21	—	152.38^b^	430.61^b^	2.83^a^	218.65^c^	231.05	209.17^c^
23	—	155.59^a^	427.55^b^	2.75^b^	223.20^b^	232.20	215.07^b^
25	—	156.19^a^	435.97^a^	2.80^a^ ^b^	224.82^a^	232.21	218.21^a^
SEM		0.74	1.58	0.02	0.76	0.91	0.84
*p*‐value		0.0009	0.0013	0.0027	<0.0001	0.5884	<0.0001
**Main effect of methionine**							
—	0.30	146.88^c^	445.17^a^	3.03^a^	211.26^c^	217.70^c^	204.82^c^
—	0.40	153.18^b^	436.82^b^	2.85^b^	219.81^b^	230.44^b^	210.23^b^
—	0.50	155.95^a^ ^b^	424.58^c^	2.72^c^	224.59^a^	235.01^a^	216.52^a^
—	0.60	159.43^a^	422.22^c^	2.65^c^	228.50^a^	238.30^a^	221.22^a^
—	0.70	158.17^a^	428.10^b^ ^c^	2.71^c^	226.97^a^	237.63^a^	217.94^a^
SEM		0.95	2.03	0.02	0.98	1.18	1.08
*p*‐value		<0.0001	<0.0001	<0.0001	<0.0001	<0.0001	<0.0001
**Protein × methionine interaction**							
21	0.30	147.26^d^ ^e^	451.03^a^	3.06^a^	209.16^e^	217.20	200.37^e^
21	0.40	150.19^c^ ^d^	439.53^b^	2.93^b^	216.14^d^	227.10	205.87^d^ ^e^
21	0.50	152.07^b^ ^c^ ^d^	419.90^e^	2.76^c^ ^d^	219.60^c^ ^d^	235.90	209.67^c^ ^d^
21	0.60	158.45^a^ ^b^	420.13^e^	2.65^e^	226.72^a^ ^b^	239.10	217.97^a^ ^b^
21	0.70	153.95^b^ ^c^	422.47^d^ ^e^	2.75^c^ ^d^	221.65^b^ ^c^	235.93	212.00^b^ ^c^ ^d^
23	0.30	147.00^d^ ^e^	430.40^c^	2.93^b^	212.36^d^ ^e^	217.97	206.97^d^
23	0.40	153.61^b^ ^c^	426.87^c^ ^d^	2.78^c^	219.77^c^ ^d^	229.87	211.87^c^ ^d^
23	0.50	155.75^a^ ^b^ ^c^	427.60^c^ ^d^	2.75^c^ ^d^	225.22^a^ ^b^	234.90	215.67^a^ ^b^ ^c^
23	0.60	159.78^a^	426.17^c^ ^d^	2.67^d^ ^e^	228.31^a^	238.93	221.40^a^
23	0.70	161.84^a^	426.73^c^ ^d^	2.64^e^	230.36^a^	239.33	219.43^a^
25	0.30	146.39^e^	454.07^a^	3.10^a^	212.26^d^ ^e^	217.93	207.13^d^
25	0.40	155.75^a^ ^b^ ^c^	444.07^a^ ^b^	2.85^b^ ^c^	223.52^b^ ^c^	234.37	212.97^b^ ^c^
25	0.50	160.04^a^	426.23^c^ ^d^	2.67^d^ ^e^	228.96^a^	234.23	224.23^a^
25	0.60	160.06^a^	420.37^e^	2.63^e^	230.46^a^	236.87	224.30^a^
25	0.70	158.71^a^	435.10^b^ ^c^	2.74^c^ ^d^	228.91^a^	237.63	222.40^a^
SEM		1.65	3.52	0.03	1.70	2.04	1.87
*p*‐value		0.0927	0.0002	0.0119	0.3380	0.4499	0.3159

*Note*: Means with different letters in each column are statistically significantly different (*p* < 0.05).

Abbrevistions: FCR: feed conversion ratio, FI: feed intake, LW: live weight; WG: weight gain.

**TABLE 5b:** Main and interactive effects of dietary crude protein and methionine levels on carcass and organ traits of Japanese quail (14–35 days of age).

Prot %	Met %	Carcass % F	Thigh % F	Breast % F	Carc % M	Thigh % M	Breast % M	Bursa M	Bursa F	Liver M	Liver F
**Main effect of protein**											
21	—	69.95	18.29^b^	27.61^c^	72.77^b^	19.67^a^	29.76	0.26^a^	0.31^a^	0.13^b^	0.25
23	—	69.95	17.20^c^	28.34^b^	72.22^b^	18.94^b^	30.02	0.21^b^	0.26^b^	0.17^a^	0.24
25	—	70.88	18.94^a^	29.53^a^	73.58^a^	19.78^a^	30.38	0.25^a^	0.31^a^	0.13^b^	0.22
SEM		0.35	0.27	0.33	0.3	0.17	0.2	0.01	0.01	0.01	0.01
*p*‐value		0.1069	<0.0001	0.0004	0.0081	0.0012	0.1073	0.0229	0.0014	0.0432	0.2001
**Main effect of methionine**											
—	0.30	69.71	17.45	27.65	72.34	19.31^b^	29.22^b^	0.27	0.46^a^	0.13	0.22
—	0.40	69.93	18.08	28.35	72.52	19.37	30.08^a^	0.23	0.22^b^	0.11	0.23^b^
—	0.50	71.21	18.91	29.11	73.09	19.59^b^	30.47^a^	0.22	0.28^b^	0.15	0.18^c^
—	0.60	70.30	18.28	28.56	72.92	18.41^c^	30.13^a^	0.28	0.25^b^	0.15	0.30^a^
—	0.70	70.16	17.98	28.79	73.41	20.62^a^	30.36^a^	0.22	0.25^b^	0.16	0.25^b^
SEM		0.46	0.35	0.42	0.39	0.22	0.26	0.01	0.01	0.01	0.02
*p*‐value		0.1809	0.0624	0.1675	0.3002	<0.0001	0.0114	0.0130	<0.0001	0.1022	<0.0001
**Protein × methionine interaction (selected key combinations shown for clarity; full data available)**											
21	0.3	67.93	17.86^b^ ^c^	25.92	72.66	18.92^b^ ^c^	28.73	0.32^a^	0.66^a^	0.17^a^ ^b^	0.33^a^
21	0.4	68.85	17.00^c^	27.47	71.94	18.96^b^ ^c^	29.46	0.25^a^ ^b^	0.16^c^	0.09^b^ ^c^	0.20^b^ ^c^
21	0.5	71.65	18.15^b^	28.44	72.29	18.97^b^ ^c^	29.99	0.17^b^	0.34^b^	0.11^b^ ^c^	0.12^c^
21	0.6	70.76	19.64^a^	27.66	73.09	20.01^a^ ^b^	30.1	0.33^a^	0.27^b^ ^c^	0.09^b^ ^c^	0.32^a^
21	0.7	70.57	18.80^a^ ^b^	28.55	73.87	21.50^a^	30.51	0.23^a^ ^b^	0.14^c^	0.20^a^	0.28^a^ ^b^
23	0.3	70.09	16.73^c^	27.73	71.6	18.05^c^	29.34	0.20^b^	0.41^b^	0.18^a^	0.21^b^ ^c^
23	0.4	69.57	18.69^a^ ^b^	28.03	72.67	19.38^b^	30.71	0.15^b^	0.18^c^	0.12^b^ ^c^	0.20^b^ ^c^
23	0.5	70.49	17.77^b^ ^c^	28.82	72.62	20.46^a^	30.57	0.31^a^	0.29^b^ ^c^	0.21^a^	0.24^b^
23	0.6	69.93	16.19^c^	28.43	72.51	16.40^d^	29.81	0.27^a^ ^b^	0.17^c^	0.18^a^	0.29^a^
23	0.7	69.68	16.61^c^	28.69	71.71	20.39^a^	29.68	0.15^b^	0.25^b^ ^c^	0.13^b^	0.27^a^ ^b^
25	0.3	71.1	17.76^b^ ^c^	29.32	72.74	20.96^a^	29.59	0.28^a^	0.32^b^	0.03^c^	0.11^c^
25	0.4	71.36	18.56^a^ ^b^	29.56	72.97	19.77^a^ ^b^	30.06	0.28^a^	0.32^b^	0.13^b^	0.31^a^
25	0.5	71.49	20.83^a^	30.08	74.35	19.34^b^	30.85	0.18^b^	0.22^c^	0.14^b^	0.18^c^
25	0.6	70.21	19.01^a^ ^b^	29.59	73.16	18.84^b^ ^c^	30.48	0.24^a^ ^b^	0.32^b^	0.19^a^	0.30^a^
25	0.7	70.23	18.54^a^ ^b^	29.12	74.66	19.97^a^ ^b^	30.9	0.28^a^	0.37^b^	0.16^a^ ^b^	0.19^c^
SEM		0.79	0.6	0.74	0.67	0.38	0.46	0.02	0.02	0.03	0.03
*p*‐value		0.1876	0.0018	0.8443	0.3512	<0.0001	0.4718	<0.0001	<0.0001	<0.0001	<0.0001

*Note*: Means with different letters in each column are statistically significantly different (*p* < 0.05).

Abbreviations: F: female, M: male.

### 4.2 Performance and Carcass Traits (14–35 Days)

Experimentally determined were the main effects of dietary protein and methionine levels and their interactions on performance and carcass traits of male and female Japanese quails (Coturnix japonica) from 14 to 35 days of age (Table 5a and 5b). A significant increase in male WG (152.38–158.49 g, *p* < 0.001), FI (430.61–444.17 g, *p* = 0.035) and live weight (218.65–228.16 g, *p* < 0.001) was observed in response to an increase in protein to 25%, while FCR remained stable (*p* = 0.280). There were small increases in female live weight (231.05–237.70 g, *p* < 0.001). Carcass, thigh and breast yields were slightly increased in males (*p* ≤ 0.090), but organ weights were not affected (*p* > 0.05).

Increasing methionine to 1.3% significantly improved most traits (*p* < 0.001), male WG reached 158.17 g, live weight 225.97 g, carcass yield 70.16% and breast yield 28.79%. The females exhibited similar trends (live weight 237.63 g and breast yield 29.69%) with increased liver weights, while bursa of Fabricius weight was not affected (p = 0.130). The highest significant interactions (*p* < 0.05) were observed at 25% protein × 1.3% methionine, with male W.G. of 161.84 g, breast yield of 29.88% (males) and 30.90% (females), and improved organ development. The optimised diet with 25% of protein and 1.3% of methionine improved growth (+6.4% WG) and breast yield (+8.3%) overall and supports sex‐tailored feeding programs in quail production. Further economic work is required.

The best fitting models for each trait during the 15 to 35 day period are summarised in Table 6. Dose‐response modelling across 21%, 23% and 25 % protein levels indicated that optimal methionine levels differed by trait, sex and protein level. The overall live weight was optimised at 0.6%, 0.37% and 0.6% (R^2^ 0.6392–0.7691), while females needed 0.61%, 0.3% and 0.7% and males 0.6%, 0.6% and 0.5%. WG was generally highest at 0.6%–0.7%. Thresholds were protein‐dependent and sex‐specific for carcass and immune organ (bursa and spleen) traits, FI and FCR responses. While R^2^ values for some carcass and organ traits were relatively low, indicating multifactorial influences, the results indicated that methionine requirements generally increased with dietary protein for growth and feed efficiency parameters, and that sex differences in carcass and immune responses were noteworthy. The nonlinear models confirmed plateau responses beyond the optimum, indicating that methionine supplementation can effectively optimise broiler performance, especially when birds are fed higher‐protein diets.

**TABLE 6:** Fitting the best model of different methionine levels on performance traits at 14–35 days of age.

Trait	Protein level %	Best model	R^2^	Equation	Optimal Met %
Live weight	21	Quadratic Broken Line	0.6392	y = 184.36 + 100.16MET + ‐56.41MET^2 (MET ≤ 0.60), y = 224.15 (MET > 0.60)	0.6
	23	Logistic 4‐parameter	0.7691	y = 233.76 + (201.63 − 233.76) / (1 + (MET / 0.37)^3.32)	0.37
	25	Quadratic	0.7413	y = 156.15 + 251.31MET + −211.05MET^2	0.6
Female live weight	21	Quadratic	0.6912	y = 154.18 + 276.13MET + −226.67MET^2	0.61
	23	Logistic 4‐parameter	0.8309	y = 242.05 + (180.24 − 242.05) / (1 + (MET / 0.26)^3.27)	0.3
	25	RNB Model 2	0.6619	y = 253.94 (1 − exp(−7.31MET)) + −22.20MET	0.7
Male live weight	21	Broken line	0.6282	y = 185.92 + 48.44MET (MET ≤ 0.60), y = 214.98 (MET > 0.60)	0.6
	23	Broken line	0.5442	y = 193.91 + 44.02MET (MET ≤ 0.60), y = 220.32 (MET > 0.60)	0.6
	25	Quadratic broken line	0.7447	y = 222.73 + −134.73MET + 275.81MET^2 (MET ≤ 0.50), y = 223.64 (MET > 0.50)	0.5
Weight gain	21	Quadratic broken line	0.4443	y = 146.68 + −10.19MET + 43.44MET^2 (MET ≤ 0.60), y = 156.20 (MET > 0.60)	0.6
	23	RNB Model 2	0.7099	y = 145.08 (1 − exp(−11.11MET)) + 24.11MET	0.7
	25	RNB Model 2	0.5996	y = 184.96 (1 − exp(−6.24MET)) + −34.11MET	0.7
Female weight gain	21	Quadratic broken line	0.5499	y = 145.95 + 10.67MET + 68.33MET^2 (MET ≤ 0.51), y = 169.53 (MET > 0.51)	0.51
	23	Logistic 4‐parameter	0.7654	y = 173.83 + (0.00 − 173.83) / (1 + (MET / 0.13)^2.43)	0.3
	25	Quadratic broken line	0.564	y = −324.98 + 2673.78MET + −3612.06MET^2 (MET ≤ 0.40), y = 166.41 (MET > 0.40)	0.37
Male weight gain	21	Quadratic broken line	0.4229	y = 147.51 + −58.10MET + 95.42MET^2 (MET ≤ 0.60), y = 147.00 (MET > 0.60)	0.6
	23	Quadratic broken line	0.4274	y = 132.44 + 29.66MET + 2.56MET^2 (MET ≤ 0.60), y = 151.16 (MET > 0.60)	0.6
	25	Quadratic broken line	0.6456	y = 167.24 + −181.04MET + 314.82MET^2 (MET ≤ 0.49), y = 153.81 (MET > 0.49)	0.49
Feed intake	21	Quadratic broken line	0.6995	y = 433.92 + 186.10MET + −430.15MET^2 (MET ≤ 0.49), y = 420.83 (MET > 0.49)	0.3
	23	Logistic 4‐parameter	0.0392	y = 426.78 + (1025.58 − 426.78) / (1 + (MET / 0.18)^9.87)	0.3
	25	Quadratic	0.5995	y = 567.71 + −500.44MET + 438.81MET^2	0.7
Feed conversion ratio	21	Broken line	0.7374	y = 3.52 + −1.51MET (MET ≤ 0.55), y = 2.70 (MET > 0.55)	0.55
	23	Logistic 4‐parameter	0.6767	y = 2.46 + (24.07 − 2.46) / (1 + (MET / 0.01)^1.12)	0.3
	25	Quadratic	0.8021	y = 4.71 + −7.20MET + 6.25MET^2	0.58
Female carcass yield	21	Quadratic broken line	0.3942	y = 76.02 + −54.12MET + 90.51MET^2 (MET ≤ 0.48), y = 70.99 (MET > 0.48)	0.48
	23	Quadratic broken line	0.0111	y = 78.21 + −43.48MET + 54.71MET^2 (MET ≤ 0.49), y = 70.03 (MET > 0.49)	0.49
	25	Logistic 4‐Parameter	0.074	y = 70.22 + (71.32 − 70.22) / (1 + (MET / 0.56)^88.81)	0.56
Female tight yield	21	Quadratic broken line	0.2673	y = 32.52 + −79.03MET + 100.55MET^2 (MET ≤ 0.54), y = 19.22 (MET > 0.54)	0.54
	23	Quadratic broken line	0.2789	y = −6.48 + 120.68MET + −144.35MET^2 (MET ≤ 0.55), y = 16.40 (MET > 0.55)	0.42
	25	Quadratic	0.2883	y = 7.06 + 49.31MET + −47.30MET^2	0.52
Female breast yield	21	Quadratic broken line	0.1714	y = 16.57 + 42.90MET + −39.10MET^2 (MET ≤ 0.49), y = 28.22 (MET > 0.49)	0.49
	23	Quadratic broken line	0.0674	y = 28.98 + −9.57MET + 17.99MET^2 (MET ≤ 0.49), y = 28.65 (MET > 0.49)	0.49
	25	Quadratic	0.0364	y = 25.74 + 16.93MET + −17.29MET^2	0.49
Female bursa fabricuos	21	Broken Line	0.8283	y = 0.94 + −2.11MET (MET ≤ 0.40), y = 0.09 (MET > 0.40)	0.4
	23	Quadratic broken line	0.0674	y = 28.98 + −9.57MET + 17.99MET^2 (MET ≤ 0.49), y = 28.65 (MET > 0.49)	0.49
	25	Quadratic	0.1435	y = 0.33 + −0.89MET + 0.85MET^2	0.7
Spleen female	21	Quadratic	0.3692	y = 0.33 + −1.17MET + 1.17MET^2	0.7
	23	Quadratic broken line	0.1563	y = 0.31 + −1.14MET + 1.44MET^2 (MET ≤ 0.55), y = 0.11 (MET > 0.55)	0.55
	25	Quadratic broken line	0.2522	y = ‐0.57 + 2.81MET + ‐2.50MET^2 (MET ≤ 0.34), y = 0.10 (MET > 0.34)	0.34
Male carcass yield	21	Quadratic	0.2003	y = 76.67 + −21.14MET + 24.71MET^2	0.7
	23	Quadratic	0.0667	y = 65.95 + 27.21MET + −27.14MET^2	0.5
	25	Quadratic broken line	0.1243	y = 78.08 + −32.82MET + 50.08MET^2 (MET ≤ 0.49), y = 74.06 (MET > 0.49)	0.49
Male tight yield	21	Logistic 4‐parameter	0.5312	y = 21.64 + (18.94 − 21.64) / (1 + (MET / 0.61)^21.78)	0.61
	23	Broken Line	0.0637	y = 14.19 + 12.88MET (MET ≤ 0.39), y = 19.16 (MET > 0.39)	0.39
	25	Quadratic	0.4434	y = 28.71 + −35.44MET + 32.53MET^2	0.7
Male breast yield	21	RNB Model 2	0.2997	y = 28.67 (1 − exp(−12.30MET)) + 2.59MET	0.7
	23	Quadratic broken line	0.2128	y = 19.46 + 49.00MET + −53.03MET^2 (MET ≤ 0.61), y = 29.68 (MET > 0.61)	0.46
	25	Quadratic broken line	0.149	y = 29.66 + −3.86MET + 12.14MET^2 (MET ≤ 0.50), y = 30.74 (MET > 0.50)	0.5
Male bursa fabricuos	21	Broken line	0.2379	y = 0.28 + −0.41MET (MET ≤ 0.42), y = 0.11 (MET > 0.42)	0.42
	23	Quadratic	0.2191	y = −0.15 + 1.09MET + −1.09MET^2	0.5
	25	Quadratic	0.1435	y = 0.33 + −0.89MET + 0.85MET^2	0.7
Spleen male	21	Quadratic	0.3692	y = 0.33 + −1.17MET + 1.17MET^2	0.7
	23	Quadratic broken line	0.1354	y = 0.54 + −2.34MET + 2.87MET^2 (MET ≤ 0.50), y = 0.08 (MET > 0.50)	0.5
	25	Logistic 4‐parameter	0.3491	y = 0.08 + (0.00 − 0.08) / (1 + (MET / 0.35)^9.66)	0.35

Abbreviations: WG: weight gain, FI: feed intake, FCR: feed conversion ratio, LW: live weight.

### 4.3 Blood Parameters

The effects of different levels of dietary protein and methionine on blood biochemical parameters in Japanese quail were investigated, and the results are presented in Table 7.

**TABLE 7:** Main and interaction effects of crude protein and methionine amino acid levels on blood parameter traits (percentage of live weight).

		Glucose	Cholesterol	Triglycerides	HDL	Hemoglobin	Urea	Ceratin	Acid uric	Albumin	Total protein	AST	ALT	T3	T4
Protein %	22	361.67	206.87	140.73	3.80	0.10	220.30	10.90	3.91	11.17	1.52	2.66	1.51	32.41	361.67
	24	367.33	204.40	118.03	3.90	0.12	226.43	10.70	3.27	12.26	1.54	2.70	1.67	36.15	367.33
	26	344.00	204.53	108.00	3.80	0.18	225.40	11.27	3.32	13.33	1.55	2.71	1.58	38.33	344.00
	*p*‐value	<.0001	0.3852	<.0001	0.9132	<.0001	0.0987	0.4199	0.0067	<.0001	0.8425	0.2617	0.0006	<.0001	<.0001
	SEM	1.36	1.42	2.04	0.19	0.00	2.14	0.31	0.15	0.22	0.04	0.02	0.03	0.25	1.36
Methionine*%*	0.70	314.72	237.33	158.11	4.28	0.14	217.78	8.56	3.46	10.80	1.42	2.61	1.80	37.59	314.72
	0.85	352.00	217.56	141.11	4.00	0.15	222.94	11.39	2.89	12.02	1.48	2.68	1.66	29.61	352.00
	1.00	353.89	215.28	98.61	5.50	0.15	226.94	11.94	3.85	12.55	1.52	2.71	1.53	39.35	353.89
	1.15	403.39	186.50	102.22	3.28	0.12	226.17	10.89	3.27	13.22	1.63	2.72	1.48	37.25	403.39
	1.30	364.33	169.67	111.22	2.11	0.11	226.39	12.00	4.03	12.68	1.63	2.73	1.47	34.35	364.33
	*p*‐value	<.0001	<.0001	<.0001	<.0001	<.0001	0.1098	<.0001	0.0008	<.0001	0.0083	0.0485	<.0001	<.0001	<.0001
	SEM	1.70	1.78	2.56	0.24	0.01	2.68	0.39	0.19	0.28	0.05	0.03	0.04	0.31	1.70
Protein*methionine interaction	22‐0.7	325.17	215.83	199.50	3.00	0.13	213.33	6.67	2.99	8.22	1.35	2.55	1.73	32.13	325.17
	22‐0.85	364.33	226.83	163.00	6.83	0.08	221.83	11.33	3.03	11.49	1.38	2.66	1.62	30.97	364.33
	22‐1	352.50	227.67	107.17	3.67	0.02	223.50	12.50	5.06	11.96	1.40	2.71	1.42	37.13	352.50
	22‐1.15	397.33	185.00	115.67	3.00	0.17	219.67	11.83	4.45	12.85	1.77	2.66	1.38	37.95	397.33
	22‐1.3	369.00	179.00	118.33	2.50	0.12	223.17	12.17	4.00	11.35	1.71	2.72	1.40	23.85	369.00
	24‐0.7	339.33	268.00	138.17	6.17	0.16	218.17	9.17	3.16	11.30	1.45	2.62	1.90	34.17	339.33
	24‐0.85	350.00	204.33	136.17	2.50	0.12	227.67	11.17	3.15	11.56	1.50	2.69	1.68	30.63	350.00
	24‐1	343.00	199.17	111.50	2.83	0.21	228.33	11.50	4.26	12.36	1.56	2.72	1.58	44.18	343.00
	24‐1.15	450.83	199.33	108.00	5.17	0.09	230.50	9.33	2.65	13.29	1.58	2.77	1.55	40.77	450.83
	24‐1.3	353.50	151.17	96.33	2.83	0.03	227.50	12.33	3.15	12.79	1.59	2.70	1.63	31.02	353.50
	26‐0.7	279.67	228.17	136.67	3.67	0.14	221.83	9.83	4.22	12.88	1.47	2.65	1.77	46.48	279.67
	26‐0.85	341.67	221.50	124.17	2.67	0.26	219.33	11.67	2.49	13.00	1.56	2.70	1.67	27.23	341.67
	26‐1	366.17	219.00	77.17	10.00	0.22	229.00	11.83	2.23	13.32	1.59	2.71	1.60	36.73	366.17
	26‐1.15	362.00	175.17	83.00	1.67	0.10	228.33	11.50	2.72	13.52	1.54	2.74	1.52	33.03	362.00
	26‐1.3	370.50	178.83	119.00	1.00	0.19	228.50	11.50	4.93	13.90	1.60	2.77	1.37	48.18	370.50
	p‐value	<.0001	<.0001	<.0001	<.0001	<.0001	0.9384	0.0176	<.0001	0.0066	0.1571	0.9445	0.4281	<.0001	<.0001
	SEM	2.78	2.91	4.18	0.39	0.01	4.37	0.63	0.32	0.45	0.08	0.05	0.06	0.51	2.78

Most blood parameters were significantly influenced by protein levels (*P* < 0.0001). Increasing dietary protein from 22% to 26% in the starter phase and 21% to 25% in the grower phase significantly reduced GLU, CHO and TG. On the other hand, the concentrations of urea, UA and TP were significantly increased with increasing protein levels, indicating higher protein catabolism and improved nitrogen status. Moderate responses of CR and Alb to protein level were observed.

Methionine levels also significantly affected most blood parameters (*P* < 0.0001 for GLU, CHO, TG, urea and UA; P < 0.01 for TP). The lowest glucose, cholesterol and triglyceride concentrations were observed at optimal methionine levels (around 0.42‐0.48%), indicating better energy and lipid metabolism. Urea and uric acid were lower in the optimal methionine treatment than in the deficient and excess treatments, indicating better amino acid utilisation and less nitrogen waste. Albumin showed less marked changes. Total protein increased with increasing methionine up to the optimum point.

Most blood parameters showed significant protein × methionine interactions (*P* < 0.0001 for GLU, CHO, TG, urea and CR; *P* < 0.05 for UA and TP). The best metabolic profile (lowest GLU, CHO and TG, and balanced urea and higher TP) was achieved with 26% protein and 0.48% methionine in the starter phase and 25% protein and 0.42% methionine in the grower phase. These interactions highlight the importance of dietary protein levels in the metabolic effects of methionine.

These results suggest that dietary protein and methionine levels and their interaction have a significant effect on metabolic efficiency and health indicators of Japanese quail. The optimised protein‐methionine combinations improved energy and lipid metabolism (lower GLU, CHO and TG) and protein status (higher TP) and nitrogen utilisation efficiency. They supported better overall physiological performance during both growth phases.

Concentrations of urea and uric acid were lower at the optimal methionine level than at deficient or excessive levels, indicating more efficient amino acid utilisation and lower nitrogenous waste. Alb showed less marked changes. TP increased with increasing methionine up to the optimum point.

## 5 Discussion

The present study provides comprehensive evidence that dietary protein and methionine levels significantly influence growth performance, carcass characteristics and blood biochemical parameters in Japanese quail. These results align with earlier research, which shows that higher CP supports tissue growth, organ development, and metabolic efficiency in young chickens (Abebe et al. 2020; Khrabrova et al. 2019; Santos et al. 2018; Ghazalah et al. 2013). The phase‐specific requirements and important protein‐methionine interactions have significant implications for commercial production optimisation.

The progressive increase of WG and FCR with increasing levels of dietary protein in the starter (WG: 58.77 to 61.20 g; FCR: 101.89 to 97.72) and grower phases is in line with the basic tenets of avian nutrition. These studies suggest that quail exhibit better tolerance and metabolic efficiency, enabling effective nutrient utilisation even with varying protein levels (Abou‐Kassem et al. 2020; Rostagno et al. 2017).

These results are consistent with previous studies in Japanese quail, which reported improved growth performance with increased protein levels (Kaur et al. 2008). The reduced FI at higher protein levels during the starter phase suggests improved nutrient utilisation from the diet, consistent with the concept of protein‐energy balance (Zhang et al. 2025). This compensatory FI adjustment demonstrates that Japanese quail possess sophisticated mechanisms for regulating nutrient intake in response to dietary composition (Lepkovsky et al. 1960).

Mathematical modelling suggested optimal methionine requirements of 0.40%–0.70%, with wide variation depending on dietary protein level and specific performance parameters. These values are generally higher than the NRC (1994) recommendations but are consistent with more recent findings on quail amino acid requirements (Hajkhodadadi et al., 2013). The significant interaction effects of protein and methionine across most performance parameters underscore the importance of the ideal protein concept (Emmert and Baker 1997; Baker 2009). The observation that optimal methionine levels generally adjusted with increasing protein levels supports the expression of amino acid requirements as ratios to dietary protein (Pesti et al., 2009).

The increase in breast muscle percentage with increasing dietary protein and optimal level of methionine is of significant economic importance because breast meat is the main marketable product. These results agree with those of Ekmay et al. (2014) and Murakami et al. (1993). However, quail focus on building overall lean mass rather than developing specific breast muscle (Alagawany et al. 2021). The negative correlation between thigh percentage and dietary protein and methionine levels indicates distinct muscle development patterns, with preferential nutrient allocation to the breast muscle (Ou et al. 2022). The demand for sulfur‐containing amino acids rises when nitrogen flow increases due to increased protein availability. This enhances metabolic efficiency and protein utilisation (Shen et al. 2014; El‐Wardany et al. 2018). The excellent FCR values obtained with optimal protein‐methionine combinations indicate the economic importance of precision nutrition in quail production (Minvielle 2004; Kidd et al. 2013).

Mechanistically, methionine supports breast muscle protein deposition through its dual role as a substrate for protein synthesis and as a regulator of the mTOR signalling pathway. As the initiating amino acid of translation and, via S‐adenosylmethionine, an important methyl donor, methionine availability is sensed by the T1R1/T1R3 amino acid receptor‐PLC‐ERK1/2 pathway, which activates mTORC1 and downstream effectors of translation initiation, while also modulating IGF‐I signalling and myogenic regulatory factors that jointly stimulate breast muscle hypertrophy and reduce muscle protein degradation (Wen et al. 2014; Zhou et al. 2016). This provides a mechanistic basis for the higher breast yields observed with methionine supplementation in the present study. The protein x methionine interaction observed for most performance traits can further be understood through the ideal protein concept, in which amino acid requirements are expressed relative to lysine, the reference amino acid for growth. Because the ratio of digestible methionine to lysine required for maximal protein accretion is relatively constant, an increase in dietary protein without a corresponding adjustment in methionine shifts this ratio away from the ideal, limiting the utilisation of the additional protein for muscle deposition; conversely, raising methionine in step with protein maintains the balanced amino acid profile needed to support the greater capacity for protein synthesis at higher protein levels, which explains why the optimal methionine level increased with dietary protein level in both phases (Emmert and Baker 1997).

Optimal nutrient levels differ between the starter and grower phases, reflecting changing metabolic priorities during development. The increased methionine requirement as a percentage of protein during the starter phase is consistent with rapid muscle development occurring during early growth (Scheuermann et al. 2003). Phase‐feeding strategies can reduce nitrogen excretion by 15%–20% without impairing performance (Rezaei et al. 2004), which is of economic and environmental concern (Kamran et al. 2008).

The fact that different traits have optimal fits provided by multiple models (quadratic, broken‐line, and logistic) shows the complex nature of biological responses to nutrients (Pesti et al., 2009). The calculated optimum methionine levels at 24%–26% protein for most performance traits are feasible formulation targets. These recommendations are justified by the better genetics of modern quail strains (Minvielle 2004) and the economic value of optimised performance. The possibility of achieving the same performance with different protein‐methionine combinations provides flexibility in feed formulation, depending on ingredient availability and prices (Kidd et al. 2013).

Increasing dietary protein and methionine levels had a significant effect on blood biochemical parameters. Raising protein levels significantly reduced GLU, CHO and TG, but increased urea, uric acid and TP. These changes are indicative of better energy and lipid metabolism and increased protein utilisation (Fouad et al. 2013; Donsbough et al. 2010). Also most blood parameters showed significant protein × methionine interactions.

Optimal supplementation with methionine further improved the metabolic profile by lowering GLU, CHO and TG and optimising nitrogenous waste products. The significant protein × methionine interactions suggested that the metabolic benefits of methionine are strongly affected by dietary protein level (Bunchasak 2009). The best metabolic profile was obtained with higher protein and an optimal methionine level in both phases.

Two mechanistic points help explain these metabolic shifts. First, the reductions in triglycerides and cholesterol with increasing methionine are consistent with methionine's role, via S‐adenosylmethionine, in suppressing hepatic lipogenesis and in supporting fatty acid oxidation: methionine supplementation has been shown to down‐regulate the lipogenic enzymes acetyl‐CoA carboxylase and fatty acid synthase, together with reduced circulating triglyceride, cholesterol, VLDL and LDL concentrations (El‐Shobokshy et al. 2022); methionine‐derived methyl groups are also required for phosphatidylcholine and carnitine synthesis, which support hepatic lipid export and mitochondrial beta‐oxidation, limiting hepatic lipid accumulation (Peng et al. 2018). Second, the glucose responses can be explained by the fact that methionine is a glucogenic amino acid: beyond the requirement for protein synthesis, methionine is catabolised by a transamination pathway that is significant mainly at high intracellular concentrations, yielding alpha‐ketobutyrate and then propionyl‐CoA and succinyl‐CoA, which can enter the TCA cycle and be diverted toward gluconeogenesis (Brosnan and Brosnan 2006). At the optimal methionine level identified in this study, methionine was preferentially channelled into protein accretion with minimal catabolic loss, whereas at deficient or excessive levels a larger proportion of methionine was likely catabolised through this route, which is consistent with the higher glucose, urea and uric acid concentrations observed away from the optimum.

An important observation in this study was that thyroid hormone concentrations responded in opposite directions to increasing dietary protein and increasing methionine. The rise in circulating T3 and T4 with higher dietary protein content is consistent with the greater metabolic demand for protein synthesis and the associated increase in basal metabolic rate: as protein intake rises, the anabolic drive for tissue accretion elevates energy expenditure, and thyroid hormones, being the principal endocrine regulators of basal metabolism and protein turnover in birds, are upregulated accordingly (Decuypere et al. 2005). By contrast, T3 declined as dietary methionine increased, which may reflect an inhibitory effect of methionine adequacy on the peripheral conversion of T4 to T3. This conversion is catalysed by the iodothyronine deiodinase enzymes (D1, D2 and D3), whose expression and activity are sensitive to nutritional and metabolic status (Darras and Van Herck 2012). A comparable inverse relationship between dietary methionine adequacy and circulating T3 has been reported previously in growing chickens, where methionine‐deficient diets were associated with elevated plasma T3 relative to methionine‐adequate controls, suggesting that methionine sufficiency normally restrains T3 production, possibly through altered deiodinase activity or reduced peripheral T4‐to‐T3 conversion (Carew et al. 2003). Beyond its role in protein synthesis, methionine also serves as the principal methyl‐group donor via S‐adenosylmethionine (SAM), supporting DNA and histone methylation reactions that regulate gene expression (Waterland 2006; Martín‐Venegas et al., 2006). Because the hypothalamic‐pituitary‐thyroid (HPT) axis is subject to epigenetic, methylation‐dependent regulation of genes encoding hypothalamic and pituitary factors as well as deiodinase enzymes, changes in methionine supply and the resulting SAM availability could modulate the sensitivity of this axis to negative feedback, thereby fine‐tuning circulating T3 concentrations somewhat independently of T4 (substrate) availability. This methylation‐mediated mechanism offers a plausible explanation for why methionine, despite promoting protein accretion in a manner similar to dietary protein, exerted an opposite effect on T3, and it highlights the hepatic and hypothalamic deiodinase pathways as a priority for future molecular investigation in quail.

The concurrent rise in AST and ALT observed at the higher dietary methionine levels should not be interpreted as a benign or inconsequential change. Because the liver is the principal site of methionine catabolism, an excess supply increases flux through the transmethylation pathway and raises hepatic homocysteine production; homocysteine accumulation (hyperhomocysteinemia) generates reactive oxygen species through autooxidation, which can damage hepatocyte membranes and increase leakage of these enzymes into the circulation (Derouiche et al. 2024). Comparable evidence in poultry indicates that excess dietary methionine can induce hyperhomocysteinemia and toxicity in several vertebrate species, including birds (Yang et al. 2020). Accordingly, the elevated AST and ALT at the higher methionine levels tested here are more appropriately interpreted as indicators of hepatic metabolic stress and an increased metabolic burden of amino acid catabolism rather than as a change without functional consequence, and methionine levels beyond the optimal range identified in this study (0.40%–0.70%) should be approached with caution in practical diet formulation.

Increasing dietary methionine decreased T3 levels (plateauing after 0.55%) and elevated liver enzymes, consistent with its roles in methylation, antioxidation and oxidative stress regulation, though excess may affect thyroid and liver responses without harm (Silva and Costa 2023; Ruan et al. 2017; Abou‐Kassem et al. 2020; Del Vesco et al. 2013). A significant protein × methionine interaction was observed on T4 (but not T3). T4 was more sensitive to nutrient levels, with the lowest values at 26% protein + 0.45% methionine and highest at 26% protein + 0.75% methionine. This reflects methionine's influence on thyroid hormone synthesis and conversion via transmethylation and trans‐sulfuration pathways (Abou‐Kassem et al. 2020; Ruan et al. 2017; Silva and Costa 2023). Methionine also selectively affected ALT (but not AST), indicating specific impacts on liver enzymes involved in amino acid metabolism (Abou‐Kassem et al. 2020; Del Vesco et al. 2013).

In conclusion, the interactive effects observed in this study highlight the importance of balanced dietary protein (24–26% in starter and 23%–25% in grower) and methionine (0.40%–0.70%) levels for optimising growth performance, carcass quality and metabolic health in modern Japanese quail strains. These findings encourage the revision of previous NRC (1994) recommendations and highlight the importance of phase‐specific and sex‐tailored feeding programs for sustainable and profitable quail production. The results should be confirmed and extended in large‐scale studies in different quail strains and commercial conditions.

## 6 Conclusions and Applications

The results of the present study demonstrated that dietary crude protein and methionine levels interact significantly to influence growth performance, carcass traits, blood biochemical parameters, thyroid hormones and liver enzyme activities in Japanese quail during both the starter (0–14 days) and grower (14–35 days) phases.

Based on the findings, the following practical recommendations can be proposed:

- **Starter phase (0–14 days)**: Diets containing 24%–26% crude protein with 0.50%–0.60% methionine provided the best combination of weight gain, feed conversion ratio, and carcass yield.
- **Grower phase (14–35 days)**: Diets containing 23%–25% crude protein with 0.50%–0.70% methionine supported superior growth performance and breast yield, with notable sex‐specific responses (females generally showed higher live weight and breast percentage).

These levels are higher than the current NRC (1994) recommendations and appear more suitable for modern, fast‐growing Japanese quail strains.

From a practical standpoint, formulating diets within the above ranges can improve growth efficiency and carcass quality. However, excessive methionine supplementation (particularly above 0.70%) should be avoided, as it was associated with elevated liver enzyme activities (AST and ALT), indicating potential metabolic stress.

Although the present study did not include a formal economic analysis, the improved feed efficiency observed with optimised protein and methionine levels suggests potential cost benefits through better nutrient utilisation. Dedicated economic evaluations considering current ingredient prices are recommended before large‐scale commercial implementation.

In conclusion, updating nutritional recommendations for Japanese quail with higher protein and methionine levels than those suggested by NRC (1994), while carefully balancing the two nutrients, can enhance productive performance and metabolic health. Future research should focus on the long‐term effects of these dietary strategies, sex‐specific nutritional requirements and comprehensive economic assessments under commercial conditions.

## Author Contributions

**Sima Moghaddaszadeh‐Ahrabi**: conceptualisation; project administration; supervision; writing – review and editing. **Ruhollah Kianfar**: conceptualisation; methodology; project administration. **Abolfazl Ghorbani**: data curation; formal analysis; writing – review and editing. **Elyas Piroz‐Maleki**: investigation; resources. **Mehdi Taginejad‐Rodbaneh**: methodology; supervision.

## Funding

Neither the authors nor their affiliated institusions have recieved any finantial or no‐nfinantial support from commercial entities that could influence the design, execution, analysis or interpretation of the data presented herein.

## Ethics Statement

All experimental procedures were reviewed and approved by the Institutional Animal Care and Use Committee of the University of Tabriz (Protocol No: IR.TABRIZU.AEC.012203.17) and carried out in accordance with established guidelines for the ethical treatment of research animals.
